# Walk your way to health: October 15 – World Walking Day

**DOI:** 10.3325/cmj.2012.53.507

**Published:** 2012-10

**Authors:** Tomislav Benjak

**Affiliations:** Croatian Institute for Public Health, Zagreb, Croatia

As defined by the World Health Organization (WHO), physical activity is “any bodily movement produced by skeletal muscles that requires energy expenditure.” It is one of the fundamental functional stimuli to the body, which, if regularly applied, reduces the risk of developing a series of chronic, primarily cardiovascular, diseases, certain types of cancer, non-insulin-dependent diabetes, and osteoporosis. Regular physical activity stimulates glucose metabolism, reduces body fat, benefits body weight regulation, lowers the blood pressure, as well as reduces the loss of bone mass. Physical activity helps to reduce stress and improves sleep quality, which is an important part of good health and life quality in general (1-4). A significant increase in the prevalence of chronic non-communicable diseases (CNCD) and outbreaks of overweight and obesity illustrate an imbalance between life style and the biological need for physical activity. Physical inactivity has thus developed into one of the major risk factors for population health and development of CNCDs. It is present in many countries and associated with 3.2 million death cases, globally involving 670 000 persons younger than 60 years, and some 30% of diabetes and ischemic heart disease cases (4,5). Epidemiological studies have also shown that physical inactivity has economical consequences, producing a major burden for the health budget. Due to all this, the WHO stresses physical activity as a vitally important segment in the CNCD prevention and recommends regular moderate exercise of minimum duration of 150 minutes weekly for healthy adults aged 18-64 years (4-6).

Different international studies reported significantly dissimilar results on the frequency of physical activity, depending on the definition of physical activity and type of the evaluation questionnaire used. A WHO study conducted in the period 2002-2003 in 51 countries across the globe, employing the shorter version of the International Physical Activity Questionnaire, showed an 18% prevalence of physical inactivity, ranging widely among men (2%-52%) and women (4%-71%) alike (7). The aspects of physical activity in Croatia were analyzed in the scope of Croatian Health Survey (CHS) 2003 and 2008. Data for 2003 showed that 30.5% of Croatian citizens found themselves physically inactive, with a similar prevalence share for men and women – 28.9% and 31.9%, respectively. In 2008, the prevalence of physical inactivity was on the upsurge at 37.7%; 36.8% in men and 38.1% women, with a significantly higher prevalence in persons over 64 years than in younger age groups. The results of both surveys also draw attention to more than 20% of physically inactive persons across all age groups (<35, 35-64, and 65+), pointing to the fact that the problem of physical inactivity exists in the overall Croatian population (8-10). A sufficient level of exercise in childhood and youth implies moderate (or intense) physical activity every day for a minimum of one hour. According to the Health Behavior in School-aged Children results for 2010, the Croatian youth is moderately active on average 4.2 days a week (4.6 days boys and 3.8 days girls) (11). According to experts from the Zagreb Faculty of Kinesiology (12), the gravest concern is that the lowest level of physical activity was associated with the 15-24 age group. What is also concerning is an increasing prevalence of modern sedentary leisure activities, such as computer games and other computer-related activities. A further intensification of such a trend can be expected due to the increasing trend of computerization. To decrease these negative trends and increase the number of physically active persons, a continuous promotion of physical activity and its benefits is needed.

Walking (hiking) is an ideal mode of daily exercise. Walking is 100% acceptable for all ambulatory individuals, but also suitable for many chronic blood pressure, heart, and asthma patients. It is safe and presents a minimum risk of injuries compared to all other physical activities. Though it is an extremely effective and healthy form of exercise, the significance of walking is rarely acknowledged, making it probably the most underestimated physical activity, lagging behind various commercial and aggressively advertized activities such as fitness, wellness, etc. The advantage of walking, compared to other forms of exercise, is that it does not need to be intense or long-term to start improving your health. The basic rule is to walk in sync with your body fitness, meaning maximally fast but not losing your breath. If gasping for breath, the rhythm should be slowed down until breathing goes back to normal, and then gradually sped up again. The purpose of walking is not to get out of breath, but rather move in tune with your physical abilities. Regular hiking in proper posture for 30 minutes five days a week has proven to reduce risk factors and benefit overall health in numerous ways. Walking prevents the risk of developing certain types of cancer (breast and colon), type II diabetes, cardiovascular diseases, as well as depression. Furthermore, it extends life expectancy even in individuals suffering from overweight or elevated blood pressure. Walking also increases mineral bone density and balances the levels of lipoproteins in the blood (4-6,13,14). After the Earth Summit in Rio de Janeiro in 1992, the Trim and Fitness International Sport for All Association proposed naming October 15 the World Walking Day. The idea was to get as many people out into the nature to develop awareness about the need for environmental protection and physical activity (walking in particular) as the simplest and most purposeful form of maintaining and improving one’s health. This year too, October 15 will be observed across the world and in Croatia. This is the ideal opportunity to start, or consolidate, one of the most health-beneficial habits.

## References

[1] Abu-Omar K (2004). Mental health and physical activity in the European Union.. Soz Praventivmed.

[2] Kohl HW (2001). Physical activity and cardiovascular disease: evidence for a dose response.. Med Sci Sports Exerc.

[3] Meisinger C, Lowel H, Thorand B, Doring A (2005). Leisure time physical activity and the risk of type 2 diabetes in men and women from the general population.. Diabetologia.

[4] World Health Organization. Physical activity. Available from: http://www.who.int/topics/physical_activity/en/ Accessed: October 16, 2012.

[5] World Health Organization. Global Recommendations on Physical Activity for Health. Available from: http://www.who.int/mediacentre/news/notes/2011/world_cancer_day_20110204/en/*.* Accessed: October 16, 2012. 26180873

[6] World Health Organization. Prevention of chronic noncommunicable diseases. datum pristupa: 09.04.2012. Available from: http://www.who.int/chp/en/*.* Accessed: October 16, 2012.

[7] Guthold R, Ono T, Strong KL, Chatterji S, Morabia A (2008). Worldwide variability in physical inactivity a 51-country survey.. Am J Prev Med.

[8] Vuletić S, Kern J. Croatian health survey 2003 [in Croatian]. Hrvatski časopis za javno zdravstvo. 2005;1:1.

[9] Marić Bajs M, Andrić A, Benjak T, Vuletić G (2012). Five-year cumulative incidence of physical inactivity in adult croatian population: the CroHort Study.. Coll Antropol.

[10] Milosevic M, Golubic R, Mustajbegovic J, Doko Jelinic J, Janev Holcer N, Kern J (2009). Regional pattern of physical inactivity in Croatia.. Coll Antropol.

[11] Kuzman M, Pavić Šimetin I, Pejnović Franelić I . The health behaviour in school-aged children 2009/10 (HBSC) [in Croatian]. Zagreb: Hrvatski zavod za javno zdravstvo; 2012.

[12] Jurakic D, Pedisic Z, Andrijasevic M (2009). Physical activity of Croatian population: cross-sectional study using International Physical Activity Questionnaire.. Croat Med J.

[13] Smoljanović T, Bičanić G, Bojanić I. On walking [in Croatian]. Available from: http://www.plivazdravlje.hr/volimhodanje/o-hodanju Accessed: October 16, 2012.

[14] Heimer S. Sports-recreational medicine – important part of public health [in Croatian]. In: Andrijašević M, Jurakić D, ed. Zbornik radova Međunarodne znanstveno-stručne konferencije “Sportska rekreacija u funkciji unapređenja zdravlja.” Zagreb: Sveučilište u Zagrebu Kineziološki fakultet, 2011.

